# To B or not to B-lines

**DOI:** 10.1186/s44158-024-00196-w

**Published:** 2024-09-05

**Authors:** Filipe André Gonzalez, Jacobo Bacariza, Joao Leote, on behalf of EchoCrit Group, Filipe Gonzalez, Rui Gomes, Rita Varudo, João Leote, Vera Pereira, Dário Batista, Vânia Brito, Corinna Lohmann, João Gouveia, Joana Manuel, Liliana Santos, Sara Lança, Lucinda Oliveira, Tiago Ferreira, Joana Ferreira, João Sampaio, José Seoane, Inês Pimenta, Cristina Martins, Ricardo Meireles, Francisco D’Orey, Maria Inês Ribeiro, Antero Fernandes

**Affiliations:** 1 Cardiovascular Research Center, Faculdade de Medicina da Universidade de Lisboa, Lisbon, Portugal; 2 Intensive Care Department, Hospital Garcia de Orta EPE, Almada, Portugal; 3 ICU in Hospital CUF Tejo, Lisbon, Portugal; 4 Escola Superior de Tecnologia da Saúde de Lisboa, Instituto Politécnico de Lisboa, Lisbon, Portugal

With great interest, we have read the paper from Boero et al. titled “Lung ultrasound among Expert operators: Scoring and Inter-rater Reliability Analysis (LESSON study), a secondary COWS study analysis from the ITALUS group” [1], which provides a focused evaluation of lung ultrasound (LUS). The author’s analysis reflects the increasing use of LUS as a valuable diagnostic and monitoring tool for assessing pulmonary conditions worldwide [2, 3]. The study is particularly noteworthy for emphasizing the inter-rater reliability of the LUS score among expert practitioners, whose assessments are crucial in determining the relevance of LUS in clinical practice [4]. By analyzing data from skilled clinicians, the article highlights the reliability of the LUS score when labeling ultrasound (US) video clips recorded from patients with COVID-19 pneumonia [5]. This focus is valuable, as skilled performance can establish benchmarks to define training standards for clinical practice through LUS assessment standardization.

Boero and colleagues [1] contribute to the expanding body of literature emphasizing the diagnostic precision of LUS, particularly when performed by trained professionals. Their findings align with recent analyses by other authors, confirming the moderate to high agreement rates achievable with LUS [6] and supporting its role in diagnosing acute lung conditions. One limitation of the study is its focus on practitioners at the expert level. While this provides valuable insights into best practices, it restricts the generalizability of the findings to a broader range of clinicians at novice and intermediate levels. This limitation is significant given that LUS is frequently utilized as a frontline diagnostic tool in diverse settings [7]. Future research should aim to broaden the participant pool to include operators with varying levels of experience. This approach would afford a more comprehensive understanding of training requirements across different clinical environments. As in cardiac ultrasound settings, Gonzalez et al. and Varudo et al. [8, 9] already showed that machine learning-enabled real-time measurements of left ventricle ejection fraction and left ventricle outflow tract velocity–time integral were strongly correlated with manual measurements, and the reproducibility was better with the machine learning system, including for novices. Besides the standardized training and interpretation that could minimize inter-operator variability among novices, using artificial intelligence (AI) tools for LUS could further improve inter-operator variability and allow less experienced users to use LUS more liberally [10].

Regarding the US system used for imaging recording, the authors used curvilinear probes with different frequencies (2–9 MHz) and different machines, including either conventional systems or ultraportable systems (i.e., Butterfly iQ) [1, 5]. In addition, the authors selected and gave, for experts’ evaluation, a proportional number of video clips recorded in each system to avoid a US system bias. However, this methodological option also promoted US imaging variability due to different lung assessment presets. Leote et al. evaluated the influence of US imaging settings on vertical artifacts (VA, used to mimic B-lines) in two phases. First, an in vitro phantom model demonstrated that variation of most of the US parameters did not significantly affect the number and scoring of optimal VA [11]. Even though artifact intensity correlated strongly with power, gain, frequency, and dynamic range, the latter increased the number of discernible VA to 3 (from 36 to 102 dB). Second, an in vivo study on 29 patients under passive invasive mechanical ventilation showed a mild influence on the VA number after controlling for physiological and operator LUS confounders [12]. As in in vitro phantoms, the dynamic range also significantly increased the VA number recognized on invasively ventilated patients (Fig. 1, note the VA identified with asterisks after increasing from 60 to 102 dB). The authors concluded that to avoid a negative impact of US settings on VA, the system preset should use a lower probe frequency (i.e., 2 to 4 MHz), with a gain and power adjusted for a value near the available upper limit (i.e., gain 90%, power − 10 dB) with a hyperechoic pleura (avoiding excessive brightness), an intermediate value of dynamic range adjusted for a discernable background media contrast (i.e., between 60 and 84 dB), without any post-processing tool such as artifact reduction, speckle reduction, frame averaging, or image enhancement. Their findings are supported by other report in the literature [13]. Regarding imaging interpretation, Boero et al. [1] variations in standard deviation results observed among clinicians were noted when distinguishing LUS score grades 0 and 1 (representing 0 to 2 B-lines or B-lines occupying less than 50% of the pleura, respectively). As described by other authors [6, 12], imaging interpretation when numbering VA is prone to some degree of inter-rater variability, even when employing a motionless probe and a controlled inspiratory volume, across three clinicians [12].

**Fig. 1:**
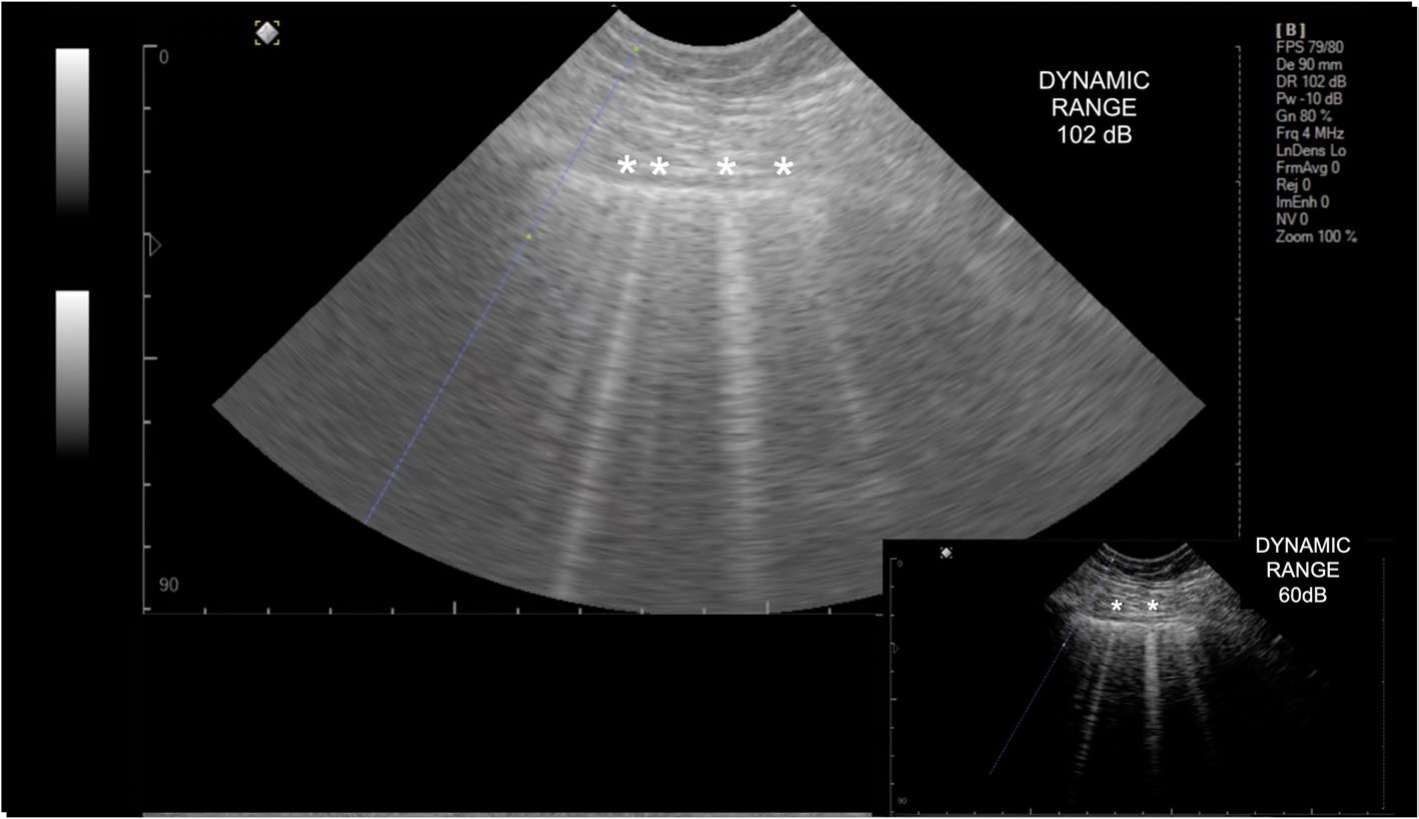
Influence of dynamic range on the number of B-lines identified (asterisk). In the bigger lung ultrasound image, more B-lines can be detected when using a dynamic range of 102 dB, compared to the smaller image at the inferior right corner, which was recorded using a dynamic range of 60 dB (four vs two B-lines)

LUS is used for diagnosis and treatment guidance, with repeated measures on the clinical evolution of a patient. If we add to the 20% inter-rater reliability in experienced users (on the LESSON study), more 15–30% for the variability of US settings (i.e., detecting 1 or 2 instead of 3 to 5 B-lines), and eventually more 10–20% among inexperienced users, we could easily reach an unacceptable inter-rater reliability. Hence, the forthcoming studies within the LUS community need to incorporate considerations for US settings [2, 7, 11–14].

As pointed by Boero and colleagues [1], LUS in clinical practice is based on a qualitative and subjective evaluation. Nonetheless, recent developments suggest using quantitative approaches to estimate the alveolar geometry after varying the probe’s center frequency [15, 16]. While the LESSON study presents a structured approach to scoring, it would benefit from a more detailed explanation of the scoring criteria. Gonzalez et al. proposed a machine learning (ML) framework (using dimensionality reduction) for automated severity analysis of COVID-19 lung ultrasounds, intended to detect frames presenting alterations indicative of the disease and provide its subsequent severity classification [17]. These results showed that the empirically used grades for LUS scores had the corresponding clinical preponderance. For example, having more B-lines in the upper lung regions in the context of a cardiogenic pulmonary edema is much more severe, as there is a cephalocaudal gradient in the edema formation, usually starting in the basal lung regions. Moreover, in some lung conditions, such as COVID-19 pneumonia, subpleural consolidations in the upper regions were associated with cardiac dysfunction and invasive mechanical ventilation [18]. Additionally, ML (support vector machine) models based on LUS were explored to improve decisions on ICU admission [19]. Using total values of LUS scores seems to result in the loss of valuable information. In fact, by leveraging ML with individual LUS findings, the prediction of ICU admission seems to be improved.

## Implications and future directions

The findings of the LESSON study have significant implications for clinical practice. By confirming the reliability of LUS among expert practitioners, the study supports integrating LUS into standard assessment protocols for pulmonary conditions. However, the LUS should move into a more precise and accurate assessment. To hit the marks, it is mandatory to decrease the inter-observer variability by standardization of LUS in clinical practice, controlling the technical parameters for patient assessment, such as type of probe, US imaging settings, and evaluating lung regions first [1, 2, 4, 14]. Also, the upcoming research should encompass a broader range of operators and address automated VA detection algorithms based on artificial intelligence-enhanced diagnostics, which may further enhance precision [20, 21].

In summary, we suggest a three-step approach for LUS standardization in clinical practice:

1. Update the current guidelines [2, 3, 14], creating a practical guide on the use of LUS and B-lines interpretation, considering the following:
  - Recommending the most appropriate US settings for each probe and the main lung disease (i.e., pneumonia, edema, fibrosis). For example, according to our studies, the most fitted US settings for B-lines detection using a curvilinear probe are as follows: mechanical index of 0.5, depth range between 10 and 12 cm (at least 6 cm below the pleura), one focal depth (on the pleura) the power of − 10 dB, gain of 90%, and equal level of time gain compensation across depth, standard line density, a dynamic range of 60 to 84 dB, center frequency of 4 MHz, without tissue equalization, or optional post-processing tools.
  - Choosing the best probe for each lung disease: a detailed image of the pleura may be needed for lung sliding or small consolidations, whereas for depth lung parenchyma, a low frequency should be selected.
  - Concise reports and inherent conclusions should also be normalized to increase the applicability of LUS across various clinical settings.
  - Defining a consensual LUS score, either global and integrating regional preponderances to grade severity (with an online tool or app to calculate it easily).
2. LUS training and interpretation recommendations to ensure basic skills and keep an acceptable inter-rater reliability (e.g., 20% as in the LESSON study):
  - A checklist of minimal training competencies for different levels of expertise.
  - List of defined specialized reference centers for training.
3. Integrating LUS AI tools where possible to standardize acquisition, measurement, and interpretation for faster and more user-friendly daily use, enabling less trained clinicians to participate.

## Data Availability

No datasets were generated or analysed during the current study.
